# Alpha-Mangostin-Rich Extracts from Mangosteen Pericarp: Optimization of Green Extraction Protocol and Evaluation of Biological Activity

**DOI:** 10.3390/molecules23081852

**Published:** 2018-07-25

**Authors:** Ali Ghasemzadeh, Hawa Z. E. Jaafar, Ali Baghdadi, Amin Tayebi-Meigooni

**Affiliations:** 1 Department of Crop Science, Faculty of Agriculture, University Putra Malaysia, Serdang 43400, Selangor, Malaysia; hawazej@upm.edu.my (H.Z.E.J.); ali_baghdadi@upm.edu.my (A.B.); 2 Department of Food Science and Technology, Tehran North Branch, Islamic Azad University, 1987973133 Tehran, Iran; amin_ir@hotmail.com

**Keywords:** response surface methodology, central composite design, α-mangostin, mangosteen, flavonoids, antimicrobial activity, antioxidant activity

## Abstract

Since α-mangostin in mangosteen fruits was reported to be the main compound able to provide natural antioxidants, the microwave-assisted extraction process to obtain high-quality α-mangostin from mangosteen pericarp (*Garcinia mangostana* L.) was optimized using a central composite design and response surface methodology. The parameters examined included extraction time, microwave power, and solvent percentage. The antioxidant and antimicrobial activity of optimized and non-optimized extracts was evaluated. Ethyl acetate as a green solvent exhibited the highest concentration of α-mangostin, followed by dichloromethane, ethanol, and water. The highest α-mangostin concentration in mangosteen pericarp of 121.01 mg/g dry matter (DM) was predicted at 3.16 min, 189.20 W, and 72.40% (*v*/*v*). The verification of experimental results under these optimized conditions showed that the α-mangostin value for the mangosteen pericarp was 120.68 mg/g DM. The predicted models were successfully developed to extract α-mangostin from the mangosteen pericarp. No significant differences were observed between the predicted and the experimental α-mangostin values, indicating that the developed models are accurate. The analysis of the extracts for secondary metabolites showed that the total phenolic content (TPC) and total flavonoid content (TFC) increased significantly in the optimized extracts (OE) compared to the non-optimized extracts (NOE). Additionally, trans-ferulic acid and catechin were abundant among the compounds identified. In addition, the optimized extract of mangosteen pericarp with its higher α-mangostin and secondary metabolite concentrations exhibited higher antioxidant activities with half maximal inhibitory concentration (IC_50_) values of 20.64 µg/mL compared to those of the NOE (28.50 µg/mL). The OE exhibited the highest antibacterial activity, particularly against Gram-positive bacteria. In this study, the microwave-assisted extraction process of α-mangostin from mangosteen pericarp was successfully optimized, indicating the accuracy of the models developed, which will be usable in a larger-scale extraction process.

## 1. Introduction

In recent years, there was an increased interest in natural sources that could provide active components to prevent the impact of free radicals on cells. For this reason, the number of studies on natural antioxidants increased considerably. Mangosteen (*Garcinia mangostana* L.) is a tree with a height of 6–25 m that belongs to the Clusiaceae family, and it is thought to originate in Southeast Asia. *Mangostana garcinia* Gaertn was approved as a synonymous name, and its vernacular names include Mangosteen (English); Manggis, Semetah, and Semontah (Malay); Dao nian zi (Chinese); and Sulambali (Tamil) [1]. The mangosteen fruit is reddish/dark purple with a juicy, soft, edible pulp and delectable taste. The pericarp of *G. mangostana* was used as a cure for chronic intestinal catarrh and dysentery, as a lotion [2], as a treatment of respiratory disorders [3], to heal skin infections and relieve diarrhea [4], and as an astringent [5]. Several biological activities were reported for the pericarp extract of *G. mangostana*, such as antioxidant [6,7], antimicrobial [8], antidiabetic [9], antiproliferative [10], and antitumor activities [11]. The biological activities of herbs/crops are related to their phytochemical constituents. The phytochemical analysis of mangosteen pericarp showed that it is rich in α-mangostin, phenolics (for example, ferulic acid, p-coumaric acid, veratric acid, t-cinnamic acid, vanillic acid, cinnamic acid, caffeic acid, mandelic acid, gentisic acid, and sinapic acid), and flavonoids (for example, epicatechin and quercetin) [7,12,13]. 

Previous studies reported that most of the biological activities of *G. mangostana* are significantly correlated with the concentration of α-mangostin [14,15]. Alpha-mangostin isolated from the extract of dried *G. mangostana* rind showed antioxidant, anticancer, and cytotoxicity activities [15]. The extraction process of α-mangostin from the mangosteen pericarp is critical [14], and the polarity and concentration of the extraction solvents were reported to be important factors. To extract different types of secondary metabolites from plant sources, various types of solvents, such as methanol, ethanol, and acetone, are commonly used [16]. The use of these organic solvents in an extraction process depends on the plant variety and the compounds targeted [16]. However, to extract on a large scale, an essential step is the optimization of the variables that are critical in the extraction process to obtain the maximal yield of the targeted compound. More useful information and optimal experimental conditions can be achieved using a good design and a suitable experimental model. Response surface methodology (RSM) was developed to optimize various extraction processes, including the extraction variables such as solvent polarity, extraction time, and temperature [17,18]. The various parameters and their interactions could be evaluated efficiently using this data analytical technology, thus reducing the experimental group number [19]. Previously, only two extraction methods (the supersonic wave and the supercritical CO_2_ method) of α-mangostin were optimized using RSM [20,21]. However, these techniques require specific equipment to extract α-mangostin. In recent years, a trans-ferulic microwave extraction method was developed for the extraction of bioactive compounds from herbs [22,23,24]. Finding a simple method with a higher extraction yield such as the microwave extraction method could be useful for extracting α-mangostin from the mangosteen pericarp on a large scale. Therefore, we are interested in the preparation of α-mangostin extracts from mangosteen pericarp using green extraction concepts. A green extraction concept is based on the design of extraction procedures that can reduce energy consumption, allow for the use of alternative safe solvents and renewable natural products, and ensure a safe and high-quality extract. To the best of our knowledge, there is no information regarding the optimization of the microwave-assisted extraction of α-mangostin from mangosteen pericarp using RSM.

This study was designed in order to enhance the extraction yield and quality of α-mangostin from the mangosteen pericarp using a green extraction method and RSM. Therefore, individual parameters such as microwave power, extraction time, and solvent polarity were optimized to extract the α-mangostin from *G. mangostana* using central composite design (CCD) and RSM. In addition, individual secondary metabolite (flavonoids and phenolic acid) profiling and the antioxidant and antimicrobial activity of the optimized extracts were evaluated.

## 2. Results and Discussion

### 2.1. Impact of Single Factors on α-Mangostin Content

**Figure 1:**
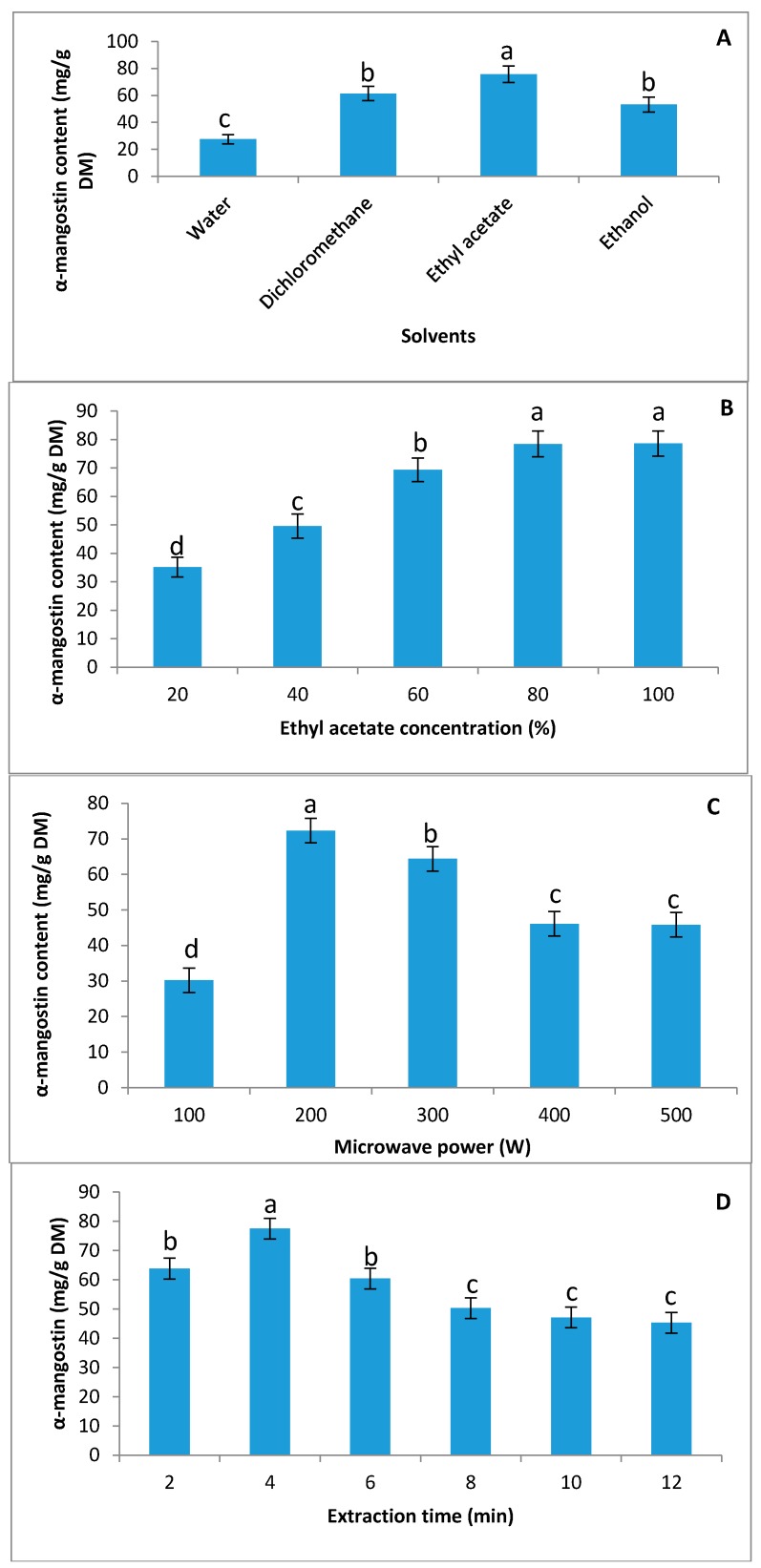
The effect of various solvents, the concentration of solvent, and microwave power on α-mangostin content in the extraction process. (**A**) Effect of various solvents on the extraction yield of α-mangostin; (**B**) effect of ethyl acetate concentration on the extraction yield of α-mangostin; (**C**) effect of microwave power on the extraction yield of α-mangostin; (**D**) effect of various extraction times on the extraction yield of α-mangostin. Different superscript lower-case letters (a,b,c,d) indicate significant differences at *p* < 0.05 (Duncan’s test).

Four green solvents, including water, ethanol, ethyl acetate, and dichloromethane, were used to extract α-mangostin from the mangosteen pericarp in this study. A significant difference between the various solvents was observed for the extraction of α-mangostin (Figure 1A). The water extract exhibited a lower concentration of α-mangostin compared to other solvents, while the highest α-mangostin value (75.66 mg/g dry matter (DM)) was observed in ethyl acetate solutions, followed by dichloromethane and ethanolic solutions (Figure 1A). No significant difference was observed in the α-mangostin concentration when the dichloromethane or ethanol solutions were used during the extraction. The results of a previous study showed that ethyl acetate (100%) was the more suitable solvent for extracting a high yield of α-mangosteen (46.2%), followed by dichloromethane (35%) [25]. Meanwhile, Bundeesomchok et al. [26] suggested ethanol and ethyl acetate for the extraction of α-mangostin from mangosteen pericarp. In this study, the highest yield of α-mangostin was obtained from the ethyl acetate extract; therefore, ethyl acetate was selected as a green solvent for the extraction of α-mangostin.

The effect of various concentrations of ethyl acetate (diluted with ethanol) on the extraction of α-mangostin was examined. A can be seen from Figure 1B, with an increase in the ethyl acetate concentration from 20–100%, the concentration of α-mangostin increased dramatically, and the highest concentrations were observed at 80% and 100% with no significant difference between them. Zhao et al. [20] reported that 67.8% ethanol exhibited the highest concentration of α-mangostin using the supersonic wave extraction method. Because there were no significant differences between 80% and 100% ethyl acetate, 80% ethyl acetate was selected for future experiments.

The influence of variable microwave power (100–500 W) on the extraction yield of α-mangostin in ethyl acetate (80% *v*/*v*) extracts is shown in Figure 1C. The minimal and maximal extraction yields were obtained at 100 W and 200 W, respectively. With increasing microwave power, the temperature will be enhanced, and following that, solvent viscosity decreases and the diffusivity increases; thus, the efficiency of extraction increases [27,28]. Hence, during microwave extraction, the moisture present in the cell matrix results in a sudden rise in local temperature due to the absorption of microwave energy [29], resulting in cell rupture. Microwave absorption also results in the increase of polyphenol solubility [30]. Therefore, the increased solubility and improved solute/solvent contact due to the rupture of the cell enhance the mass transfer from a solid to liquid phase. This mechanism works within the boundaries of the microwave’s power. Any future increase in microwave power may lead to opposite results [28]. Also, a future increase in microwave power could damage the molecular structure of the targeted compound in the extract [29]. The results of this study showed that an additional increase in microwave power from 200 W to 500 W resulted in a significant decrease in the α-mangostin content. Additionally, no significant difference in the α-mangostin content was observed between 400 W and 500 W. Therefore, 200 W was chosen for future experiments. The precipitate extraction of secondary metabolites by increasing microwave power is related to the energy of microwaves on bio-molecules inducing ionic conduction and dipole rotation, resulting in power dissipating inside the solvent and plant material, ultimately leading to the generation of molecular movement and heating [31].

It is essential to economize the cost of the α-mangostin extraction process while reducing/minimizing the extraction time. In this study, a range of 2–12 min was examined for the recovery of α-mangostin using the extraction process (Figure 1D). With an increase in extraction time from 2 to 4 min, the α-mangostin value increased significantly. A further increase in the extraction time to 12 min resulted in a decrease in the α-mangostin value; however, there was no significant difference between 8, 10, and 12 min. Xiao et al. [32] reported that the highest content of flavonoid from *Radix astragali* using microwave extraction was obtained at 25 min, while a further increase in extraction time resulted in decreasing flavonoid content. From these results, it was concluded that the α-mangostin extraction from mangosteen pericarp was approximately complete within 4 min. A longer extraction time could damage the molecular structure of α-mangostin, resulting in a decrease in concentration. Therefore, 4 min was chosen for future study.

### 2.2. Model and Response Surface Analysis

The selected levels of extraction variables (time, microwave power, and the percentage of solvent) were used for the experimental design after the preliminary investigations. Central composite design (CCD) and RSM were used for the regression and response surface analyses.

### 2.3. Model Fitting

**Figure 2:**
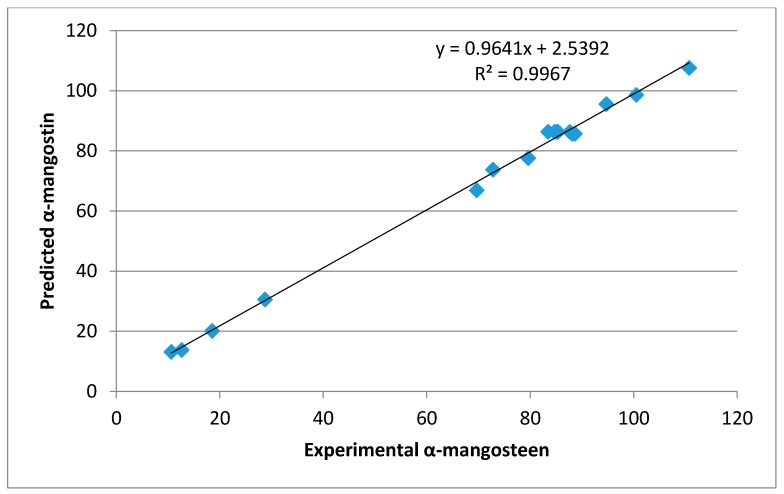
The regression of predicted and experimental values of α-mangostin.

An RSM approach was employed to determine the effects of extraction conditions, including time (min; X_1_), microwave power (W; X_2_), and ethyl acetate concentration (*v*/*v* %; X_3_), on the extraction of bioactive compounds from mangosteen pericarp cultivated in Malaysia. The α-mangostin concentration of the extracts is reported in Table 1. The highest concentration of α-mangostin (116.8 mg/g DM) was observed at an extraction time of 3 min, a microwave power of 150 W, and 70% ethyl acetate. The experimental data were fitted to a second-order polynomial model. Table 2 illustrates the regression coefficients of the model, which were considered significant (*p* < 0.05). In fact, the statistical analysis showed that microwave power and ethyl acetate concentration had a significant effect on the α-mangostin concentration. No significant effect of extraction time was observed for α-mangostin content in the extract. In addition, the results showed that the microwave power of extraction and ethyl acetate concentration resulted in a positive quadratic effect on the response. It is clear that there was a significant interaction between the microwave power and the ethyl acetate concentration parameters on the α-mangostin concentration. Table 2 summarizes the validity of the model that was examined using lack-of-fit testing. An ANOVA for the lack-of-fit test for the α-mangostin response was not significant, confirming that the model adequately described the experimental data. In addition, a satisfactory determination coefficient (R^2^) of 0.983 was obtained for α-mangostin, which represented an excellent correlation between the independent factors and the response. A higher R^2^ value indicates a better fit of the experimental model to the real data. Alternatively, a lower the value of R^2^ signifies a lower correlation; however, this can elucidate the behavior of independent variables. As shown in Table 1, the experimental value of the α-mangostin content was close to the predicted value, and there was no significant difference between the predicted and experimental values. An analysis of the regression (Figure 2) also showed that the predicted value of α-mangostin was correlated linearly and significantly with the experimental value (R^2^ = 0.9967).

The second-order polynomial equation was generated for the α-mangostin response, and it revealed the functional relationship between the factors, regardless of their significance. The predicted models developed for α-mangostin extraction from mangosteen pericarp (Y) were as follows:

Y = 350.79850 − 1.52830X_1_ − 1.029578X_2_ + 0.50126X_3_ − 0.019450X_1_X_2_ + 5.25500X_1_X_3_ + 0.012480X_2_X_3_ + 0.030680X_1_^2^ + 0.12136X_2_^2^ − 0.10180X_3_^2^ (1)

After three revisions of the regression equation and the elimination of non-significant items (*F*-value < *F*-critical value; *p* > 0.05), the predicted models established for α-mangostin content (Y) were modified to be

Y = 350.79850 − 0.1029578X_2_ + 0.50126X_3_ + 0.012480X_2_X_3_ + 0.12136X_2_^2^ − 0.10180X_3_^2^ (2)

According to the analysis of variance, the model with a good coefficient of determination (R^2^ = 0.983) for Y was significant (*F*-value = 0.0016), which implied that the three factors influenced the extraction efficiency of α-mangostin. The C.V. expressed the standard deviation as a percentage of the mean, and it was found to be 3.481% (<5.00%) for the α-mangostin yield, implying that the models were reproducible.

The lack-of-fit test was not significant, which indicates an adequate fit of the models to the experimental data for all the response variables. The model is valid if the ratio of the mean square of the lack of fit to the pure error is smaller than the tabulated one. Therefore, the lack-of-fit statistic would be non-significant (*p* > 0.05). The results obtained showed that the ratio of the mean square of the lack of fit to the pure error was smaller than the tabulated value. The *p*-value obtained was 0.1328, which was superior to 5%. Thus, the model was deemed valid.

### 2.4. Response Surface Analysis of the α-Mangostin Concentration in Mangosteen Pericarp Extracts

**Figure 3:**
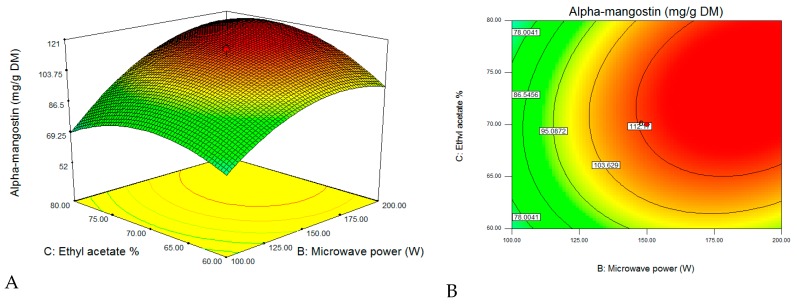
The response surface plots for the effects of microwave power (W) and ethyl acetate concentration (%) on the α-mangostin content of *G. mangostana*. (**A**) Three-dimensional (3D) view; (**B**) flat view.

**Figure 4:**
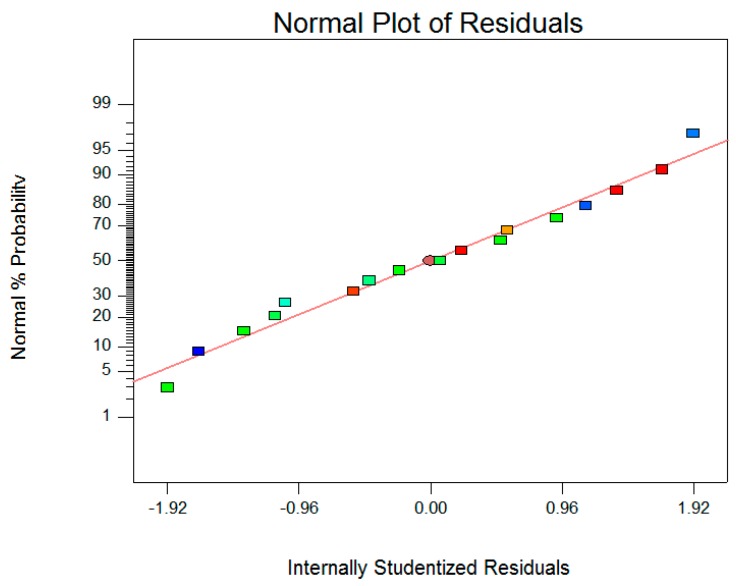
The normal plot of residuals.

After validating the model, three-dimensional response surfaces (Figure 3) were plotted, and a normal plot of residuals (Figure 4) was generated for the α-mangostin concentration against the two significant combined factors, including microwave power and ethanol concentration, while the third factor was set to be constant at a level (−1) corresponding to 40 min. As clearly shown from Figure 3, the α-mangostin concentration increased with increasing microwave power for the extraction and decreasing ethanol concentration. The corresponding area (red color) is extremely large, and it represents a value that can be close to 100% when the extraction time was set at 40 min. Ethyl acetate is soluble in water, and it is a sufficiently polar solvent to be heated using microwave energy. The polarity of the solvent is very important during microwave-assisted extraction procedures. Therefore, the mixture of water with alcohol makes it a moderately polar solvent to ensure the maximum extraction of secondary metabolites [33]. Furthermore, there is a general “like dissolves like” principle that explains that different solvents only extract phytochemicals/secondary metabolites that share a similar polarity. Polar solvents possess the ability to absorb more microwave energy because of their high dielectric constant [33,34]. Even the completely dried plant cells contain traces of moisture that are targeted by microwave energy. In the microwave extraction method, water molecules present within the plant texture quickly absorb microwave energy, causing a significantly higher temperature inside cells [35]. With increasing internal temperature, cell disruption is facilitated by internal superheating that further induces the desorption of phenolic compounds from the texture with an enhanced recovery rate into the surrounding solvent. Pan et al. [36] reported that an increasing internal temperature in the plant cell resulted in the rupture of cell walls, followed by the release of compounds into the surrounding solvent. With microwave extraction, the energy transfer occurs via two mechanisms, specifically, via dipole rotation and ionic conduction, by means of reversals of dipoles and the displacement of charged ions present in the solute, as well as in the solvent [37]. The radiation frequency corresponds to the rotational motion of molecules; in condensed matter, energy absorption immediately causes energy redistribution between molecules and homogeneous heating of the medium. In fact, there is a migration of dissolved ions which increases the penetration of the solvent into the matrix, thus facilitating the collection of the target compounds [38].

### 2.5. Optimization and Prediction of Parameters

After applying a uniform design which effectively narrowed the range of extraction conditions, some of the sophisticated tests were investigated in succession using orthogonals to obtain more efficient results. To maximize the α-mangostin content in the mangosteen pericarp extract, the extraction conditions were optimized. Multiple regression analysis was used to achieve the optimal extraction conditions. The predicted models were used to optimize the extraction process based on the highest desirability values after the regression analysis. The optimal variables for the extraction of α-mangostin from the mangosteen pericarp were obtained using the Design Expert software, and they are shown in Table 3. The highest α-mangostin content for a pericarp extract of mangosteen (121.01 mg/g DM) was predicted at a microwave power of 189.20 W, an ethyl acetate percentage of 72.40% (*v*/*v*), and an extraction time of 3.16 min.

### 2.6. Optimal Condition Validation

Experiments were performed using the optimized conditions to validate the models. As seen in Table 3, the experimental values (120.68 mg/g DM) of α-mangostin were similar to the predicted values, and there were no significant differences between the predicted and experimental values. This result indicates that the individual models developed for the α-mangostin concentration were suitable to efficiently optimize the extraction conditions. Thus, this method was accurate, reliable, and reproducible.

### 2.7. Polyphenolic Compound Determinations of Optimized and Non-Optimized Extracts

Mangosteen peel was reported to contain phenolic compounds [39]. The concentrations of phenolic compounds in the different organs of mangosteen varied depending on the extraction technique, the type of solvent, the maturity of fruit, and the drying process [12,40]. In this study, the range of phenolic concentrations differed. The total phenolic content (TPC) of the optimized extract was found to be 368.2 ± 21.06 mg gallic acid equivalent (GAE)/100 g DM, which was approximately 18.1% more than that of the non-optimized extract (311.72 ± 19.55 mg GAE/100 g DM), as shown in Table 4. In addition, the total flavonoid content (TFC) was found to be 279.19 ± 19.55 mg quercetin equivalent (QE)/100 g DM, which was approximately 45.03% more than the non-optimized extract (192.5 ± 17.28 mg QE/100 g DM). The recovery of the total flavonoid content (TFC) was higher than that of the TPC with the optimization of the extract. A total of three phenolic compounds and three flavonoid compounds were successfully identified in the optimized and non-optimized extracts, including trans-ferulic acid, cinnamic acid, caffeic acid, rutin, catechin, and quercetin. The quantitative results (Table 4) of the optimized extract showed that trans-ferulic acid was the primary compound, and cinnamic acid was the second most abundant compound followed by catechin. Zarena and Sankar [7] reported catechin and quercetin as the main identified flavonoids in mangosteen pericarp. Also, cinnamic acid, ferulic acid, sinapic acid, and syringic acid were identified as main phenolic compounds in mangosteen pericarp. Zadernowski et al. [12] reported protocatechuic acid as the abundant phenolic acid in peel extract. In our study, sinapic acid and syringic acid were not detected from the extracts. The production of secondary metabolites in the plants may vary because of various reasons, including varietal difference, climate changes, agricultural practices, etc. [41,42,43]. The optimization process significantly improved the amount of individual phenolic acids and flavonoids except for that of quercetin.

### 2.8. Antioxidant Activity of Optimized and Non-Optimized Extracts of Mangosteen

**Figure 5:**
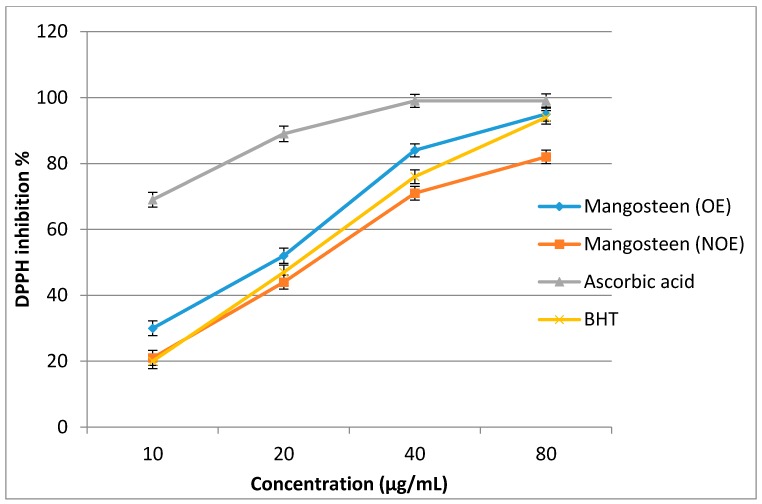
The 2,2-diphenyl-1-picryl-hydrazyl-hydrate (DPPH) activity of optimized extract (OE) and non-optimized extract (NOE) from mangosteen pericarp. Bars indicate standard errors of the means.

Optimized and non-optimized extracts from the mangosteen pericarp were tested for antioxidant properties using the 2,2-diphenyl-1-picryl-hydrazyl-hydrate (DPPH) assay. The extracts were tested at concentrations ranging from 10 to 80 μg/mL. As shown in Figure 5, the DPPH activity increased significantly with an increase in the concentration of the extracts. The optimized extracts exhibited higher DPPH activity compared to the non-optimized extracts. The optimized extract (OE) presented a lower half maximal inhibitory concentration (IC_50_ = 20.64 µg/mL) compared to that of the non-optimized extract (NOE; 28.50 µg/mL), which indicates potent antioxidant properties. The result of a recent study showed that the extract of *G. mangostana* has antioxidant activity with an IC_50_ value of 30 µg/mL compared to Butylated hydroxyanisole (BHA), (IC_50_ = 20.0 µg/mL) using the DPPH assay [7]. In another study, α-mangostin isolated from an extract of dried *G. mangostana* rind showed an antioxidant activity with an IC_50_ value of 7.4 µg/mL compared to ascorbic acid (IC_50_ = 4.5 µg/mL) using the DPPH assay [44]. Ascorbic acid as a positive control showed the highest antioxidant activity (IC_50_ < 10 µg/mL) compared to both the OE and NOE; however, more interestingly, the antioxidant activity of the OE was higher than that of Butylated hydroxytoluene (BHT), (second positive control). In addition, the NOE had less antioxidant activity than that of BHT. As can be seen from Table 5, the OE also exhibited higher ferric reducing antioxidant potential (FRAP) activity (497.42 ± 12.73 μM of Fe (II)/g DM) compared to that of the NOE (344.60 ± 8.61 μM of Fe (II)/g DM). The OE had higher FRAP activity than BHT, but one that was lower than that of ascorbic acid. In general, the antioxidant activity of the mangosteen pericarp extracts improved after the optimization of the extraction process. Due to the higher amount of phytocompounds, the mangosteen extracts possessed superior radical-scavenging activities. The results of several studies indicated that the free-radical-scavenging power of the mangosteen pericarp was significantly correlated with the concentration and type of phytochemicals [6,45,46]. After collecting more data, especially data from the industry, the models should be renewed. Subsequently, the design space should also be recalculated to result in a more reliable and flexible pharmaceutical process.

### 2.9. Antimicrobial Activity of Optimized and Non-Optimized Extracts

The inhibitory effects of the OE and NOE of mangosteen pericarp on test pathogens are shown in Table 6. The antibacterial activities of the mangosteen pericarp extract were significantly different (*p* < 0.05) from those of the NOE and OE with inhibition diameter (ID) values ranges of 10–18 mm and 6–14 mm, respectively, against the seven test pathogens. The OE exhibited the highest antibacterial effects against Gram-positive bacteria compared to Gram-negative bacteria. Among the bacterial strains studied, *Staphylococcus aureus* and *Mycobacterium smegmatis* were more sensitive to the mangosteen pericarp extracts. More interestingly, the OE showed strong antibacterial activity against *Staphylococcus aureus* (ID: 18 mm) compared to ciprofloxacin (ID: 16 mm)*.* The result of previous studies on the NOE of mangosteen pericarp showed antibacterial activity against *Escherichia coli*, *Staphylococcus aureus*, *Salmonella typhi*, *Shigella dysenteriae*, *Klebsiella pneumoniae*, and *Vibrio cholera* [8,47,48]. Alpha-mangostin was reported to be a potent agent against various ranges of bacterial strains. The observed antibacterial efficiency of the studied extracts may be due to their phytochemical composition. Indeed, the OE of mangosteen pericarp exhibited high amounts of bioactive phenolic, flavonoid, and α-mangostin (120.68 mg/g DM). Therefore, the antibacterial activity of the optimized extract could be related to the concentration of α-mangostin or polyphenols in the extracts. To the best of our knowledge, this is the first report of the antimicrobial activity of optimized mangosteen pericarp extract against these pathogens, except for *S. aureus* and *E. coli* [8].

## 3. Materials and Methods

### 3.1. The Sampling of the Mangosteen Fruit

Mangosteen fruits were harvested from a mangosteen farm located in Johor, Malaysia. All harvested fruits were washed with pure water. The pericarps of the fruits were separated and dried in a 45 °C oven for five days. The dried pericarps were powdered using a grinder (0.355 mm) and were sieved (80 mesh). The samples were kept at −20 °C for future analysis.

### 3.2. Extraction Parameters and Preliminary Study of Extraction Parameters

Extraction was conducted using a microwave extractor (Multivalve 3000, Graz, Austria). Specifically, 2 g of powdered mangosteen pericarp was extracted with 20 mL of green solvent (water, ethanol, ethyl acetate, or dichloromethane). The microwave power during extraction was adjusted using a microwave power control panel. An extraction time of 2–12 min, a microwave power of 100–500 W, and solvent percentages of 20–100% (*v*/*v*) were chosen as the variables for the extraction process. A one-factor-at-a-time method was used to investigate the influence of each factor on the targeted yield in extracts. The effect of different concentrations of ethyl acetate (diluted with ethanol) on the extraction yield was also evaluated. The extracts were filtered through Whatman No. 1 paper, transferred to Falcon tubes, and kept at −20 °C for future analysis. Experiments were carried out in triplicate to ensure reproducibility.

### 3.3. RSM Analysis

RSM is an experimental statistical technique applied to the multiple regression analysis using quantitative data obtained from properly designed experiments. Various parameters that influenced the extraction efficiency were optimized to efficiently extract active compounds, including phenolic acids and flavonoids, from pigmented rice bran. In this study, the relationships among time (X_1_), microwave power (X_2_), and solvent percentage (X_3_) were investigated using CCD to obtain the optimal extraction conditions. The quadratic polynomial step-by-step regression method and data were analyzed using the Design Expert (Version 7, Stat-Ease, Inc., Minneapolis, MN, USA) software. The model shown below was used to predict the response variables.

Y = b_0_ + b_1_X_1_ + b_2_X_2_ + b_3_X_3_ + b_1_^2^X_1_^2^ + b_2_^2^X_2_^2^+ b_2_^3^X_2_^3^ + b_1_b_2_X_1_X_2_ + b_1_b_3_X_1_X_3_ + b_2_b_3_X_2_X_3_, (3)

where Y is the predicted dependent variable; b_0_ is a constant that fixes the response at the central point of the experiment; b_1_, b_2_, and b_3_ are the regression coefficients for the linear effect terms; b_1_b_2_, b_1_b_3_, and b_2_b_3_ are the interaction effect terms; and b_1_^2^, b_2_^2^, and b_3_^2^ are the quadratic effect terms. The regression coefficients of the individual linear, quadratic, and interaction terms were determined according to an analysis of variance (ANOVA). To visualize the relationship between the response and experimental levels of each factor and to deduce the optimal conditions, the regression coefficients were used to generate three-dimensional (3D) surface plots and contour plots from the fitted polynomial equation. The factor levels were coded as −1.682, −1, 0, +1, and +1.682. The variables were coded as described by the following equation:

(4) $$Xi=\frac{Xi-X0}{\Delta X},$$

where *X_i_* is the (dimensionless) coded value of the variable *X_i_*, *X*_0_ is the value of *X* at the central point, and Δ*X* is the step change.

### 3.4. HPLC Analysis of α-Mangostin

Alpha-mangostin in the extracts was identified using an Agilent HPLC 1200 system (Agilent Technologies, Santa Clara, CA, USA). The separation was conducted at 25 °C on a Lichrocart column (5 μm, 4 mm × 250 mm). The mobile phase for the method developed consisted of acetonitrile (solvent A) and 0.2% aqueous formic acid in water (solvent B). The method employed a step-wise linear gradient. In addition, the injection volume and flow rates were 20 μL and 1 mL/min, respectively. The UV wavelength was set at 240 nm. The calibration curve of α-mangostin was performed at different concentrations (15, 30, 60, 120, and 240 μg/mL). The amount of α-mangostin was calculated based on a linear equation: Y = 30871.46X + 1941.82, R^2^ = 0.9983. Each calibration point was conducted in triplicate.

### 3.5. HPLC Analysis of Phenolics and Flavonoids

Qualitative and quantitative analysis of the samples was performed using an Agilent HPLC 1200 system (Agilent Technologies, Santa Clara, CA, USA). A C18 column with ZORBAX (5 μm, 2.1 mm × 12.5 mm) was equipped. The mobile phase for the method developed consisted of 0.03 M ortho-phosphoric acid (solvent A) and HPLC-grade methanol (solvent B). The method employed a step-wise linear gradient. The column was maintained at 35 °C. In addition, the injection volume and flow rates were 10 μL and 1 mL/min, respectively. A standard solution of each compound was prepared at different concentrations, and a calibration curve was prepared. Linear equations of each compound were as follows: gallic acid (Y = 872.62X + 119.20), trans-ferulic acid (Y = 594.39X + 85.46), cinnamic acid (Y = 294.50X + 60.29), caffeic acid (Y = 317.69X + 57.03), quercetin (Y = 314 X + 86.29), catechin (Y = 438.11X + 106), and rutin (Y = 297.36X + 84.25).

### 3.6. Evaluation of Antioxidant Activity

#### 3.6.1. DPPH Assay

The optimized pericarp extracts of mangosteen were examined for their hydrogen-donating ability toward DPPH, which is a stable free radical. The sample extracts and ascorbic acid were adjusted to 100 μL with 3 mL of 0.1 mM DPPH in methanol and vortexed well. The solutions were incubated in the dark for 30 min. The scavenging activities of the extracts were determined from the absorbance at 517 nm against methanol as a blank solution [41]. The following formula was used to calculate the scavenging activity:

% inhibition = (absorbancecontrol − absorbancesample)/absorbancecontrol × 100(5)

#### 3.6.2. Ferric Reducing Antioxidant Potential (FRAP) Assay

The FRAP assay was used to evaluate antioxidant activity. Briefly, 200 µL of the extracts were mixed with 2.0 mL of FRAP reagent (pH = 3.6). The mixture was incubated in a water bath at 25 °C for 30 min. The absorbance of the solution (blue color) was measured against acetate buffer (the blank) at 593 nm. A standard curve was prepared using concentrations of 100–1000 mM of FeSO_4_ × 7 H_2_O. The results are expressed in μM of Fe (II)/g DM [41].

### 3.7. Antibacterial Test

Five reference bacterial strains and two laboratory strains from our laboratory stock culture confirmed to be multidrug-resistant bacteria were used for the antibacterial assay. The reference and laboratory strains are four Gram-positive bacteria (*S. aureus* (NCBI 50080), *M. smegmatis* (ATCC 700084), *L. ivanovii*, (ATCC 19119), and *S. uberis* (ATCC700407)), and three Gram-negative bacteria (*E. cloacae* (ATCC 13047), *E. coli* 180, and *V. parahaemolyticus* (ATCC 17802)) that were reported to be resistant to sulphamethoxazole, ampicillin, streptomycin, cefuroxime, cephalexin, tetracycline, and nalidixic. They were tested against the mangosteen optimized and non-optimized extracts.

The bacteria were cultivated in Mueller-Hinton broth at the appropriated temperature (34–37 °C) of the strains. Then, the turbidity of each culture of bacterium was adjusted to reach 1–5 × 10^8^ colony-forming units (CFU)/mL. Briefly, 100 μL of a suspension containing 10^8^ CFU/mL of bacteria cells was spread on Petri plates. The paper discs (6 mm in diameter) were separately impregnated with 20 μL of the extract (100 μg/mL) of mangosteen pericarp and placed on an agar plate which was previously inoculated with the selected test microorganisms. Ciprofloxacin was used as a positive reference for the bacteria. Discs without samples were used as a negative control. Plates were kept at 4 °C for 1 h. The inoculated plates were incubated at 37 °C for 24 h. The antimicrobial activity was assessed by measuring the diameter of the growth in millimeters (including disc diameter of 6 mm) for the test organisms compared to the controls [49].

### 3.8. Data Analysis

The data were analyzed using the SAS (Statistical Analysis System) Version 9.2 software and Duncan’s multiple range test with significance set at the *p* < 0.05 level. The mean and standard deviation (*n* = 3) of each standard and sample were calculated.

## 4. Conclusions

This study investigated the optimization of the extraction process of α-mangostin from mangosteen pericarp using a microwave extraction method with ethyl acetate as a green solvent. A central composite design (CCD) was successfully employed to determine the optimal extraction conditions to obtain a high-quality extract with potential antioxidant and antimicrobial activities. The optimal microwave power, time, and ethyl acetate percentage of extraction to maximize the α-mangostin extract from mangosteen pericarp (120.68 mg/g DM) were 189.20 W, 3.16 min, and 72.40% (*v*/*v*), respectively. The OE of the mangosteen pericarp exhibited higher concentrations of TPC, TFC, and individual flavonoids and phenolic acids than the NOE. Trans-ferulic acid was found to be an abundant phenolic compound. In addition, the free-radical-scavenging power of the mangosteen pericarp extract obtained under optimal conditions was higher than that of the NOE. The OE exhibited the highest antibacterial activity, particularly against Gram-positive bacteria. This study is the first report of the optimization of the microwave-assisted green extraction process of α-mangostin from mangostin pericarp, and it provides a significant basis to further investigate the separation of this effective natural substance.

## Figures and Tables

**Table 1:** The experimental design and response of three independent variables on α-Mangostin content.

Run	Independent Variables			α-Mangostin Content (mg/g DM)	
	Time (min)	Microwave Power (W)	Ethyl Acetate (%)	Experimental	Predicted
1	2.0	200.0	60.0	77.6	79.1
2	1.3	150.0	70.0	81.5	82.4
3	3.0	150.0	86.8	90.4	90.8
4	3.0	66.0	70.0	50.7	52.1
5	3.0	150.0	53.1	80.1	84.6
6	3.0	150.0	70.0	98.8	99.5
7	4.6	150.0	70.0	101.5	102.7
8	4.0	200.0	80.0	110.5	113.8
9	4.0	100.0	60.0	74.3	75.9
10	2.0	200.0	80.0	100.6	102.1
11	3.0	150.0	70.0	115.7	116.5
12	3.0	150.0	70.0	116.8	115. 9
13	2.0	100.0	60.0	46.6	47.2
14	3.0	150.0	70.0	116.4	115.0
15	3.0	150.0	70.0	115.7	115.4
16	2.0	100.0	80.0	52.5	54. 9
17	4.0	200.0	60.0	93.6	94.6
18	3.0	234.0	70.0	106.4	105.8
19	4.0	100.0	80.0	62.9	64.2
20	3.0	150.0	70.0	115.4	116.0

**Table 2:** The analysis of variance for the experimental results of α-mangostin content from mangosteen pericarp extracts.

Source	Sum of Squares	df	Mean of Square	*F*-Value	*p*-Value (Prob > F)
Model	7699.587	9	855.5097	12.25099	0.0016 **
X_1_	72	1	72	1.031047	0.3437
X_2_	607.7841	1	607.7841	8.703531	0.0214 *
X_3_	2931.865	1	2931.865	41.98461	0.0003 **
X_1_X_2_	34.04723	1	34.04723	0.48756	0.5075
X_1_X_3_	27.61503	1	27.61503	0.39545	0.5494
X_2_X_3_	350.4384	1	350.4384	5.018314	0.0401 *
X_1_^2^	39.6321	1	39.6321	0.567536	0.4758
X_2_^2^	3139.328	1	3139.328	44.9555	0.0003 **
X_3_^2^	606.7706	1	606.7706	8.689018	0.0215 *
Residual	488.8233	7	69.8319		
Lack of Fit	351.762	3	117.254	3.421943	0.1328 ^n.s^
Pure Error	137.0613	4	34.26533		
R^2^				0.983	
Adj R^2^				0.970	
CV%				3.481	
Cor Total	8188.41	16			

* and ** indicate significance at *p* < 0.01 and *p* < 0.05, respectively; ^n.s^: non-significant.

**Table 3:** The predicted and experimental values of α-mangostin obtained under the optimal extraction conditions.

Microwave Power (W)	Time (min)	Solvent Percentage (%)	Desirability	α-Mangostin (mg/g DM)	
				Predicted	Experimental
189.20	3.16	72.40	1	121.01	120.68

**Table 4:** Total phenolic content (TPC), total flavonoid content (TFC), and individual phenolics and flavonoids from optimized and non-optimized extracts of *Garcinia mangostana*.

Secondary Metabolites	Optimized	Non-Optimized
TPC	368.2 ± 21.06 ^a^	311.72 ± 19.55 ^b^
trans-ferulic acid	148.91 ± 17.69 ^a^	112.41 ± 16.53 ^b^
cinnamic acid	82.54 ± 9.21 ^a^	56.74 ± 9.21 ^b^
caffeic acid	55.06 ± 6.25 ^a^	41.42 ± 6.18 ^b^
TFC	279.19 ± 19.55 ^a^	192.5 ± 17.28 ^b^
rutin	34.73 ± 7.06 ^a^	30.16 ± 5.44 ^b^
catechin	78.61 ± 9.18 ^a^	42.71 ± 5.92 ^b^
quercetin	40.15 ± 8.55 ^a^	36.22 ± 6.11 ^a^

Unit of TPC: mg gallic acid equivalent (GAE)/100 g DM; unit of individual phenolic acids: mg/100 g DM. Unit of TFC: mg quercetin (QE)/100 g DM; unit of individual flavonoids: mg/100 g DM. Different superscript lower-case letters in each row indicate significant differences at *p* < 0.05 (Duncan’s test).

**Table 5:** The ferric reducing antioxidant potential (FRAP) activity of optimized extract (OE) and non-optimized extract (NOE) from mangosteen.

Samples	FRAP (μM of Fe (II)/g DM)
NOE	344.60 ± 8.61 ^d^
OE	497.42 ± 12.73 ^b^
Ascorbic acid	783.27 ± 16.28 ^a^
BHT	421.91 ± 10.33 ^c^

Different superscript lower-case letters in each row indicate significant difference at *p* < 0.05 (Duncan’s test).

**Table 6:** The antimicrobial activity of optimized and non-optimized extracts from mangosteen pericarp; ID: diameter of inhibition (mm).

Bacterial Strains	ID (mm)			
	Negative Control	Positive Control	OE	NOE
*Listeria ivanovii*	−	18	17	14
*Staphylococcus aureus*	−	16	18	14
*Mycobacterium smegmatis*	−	17	16	12
*Streptococcus uberis*	−	18	14	14
*Vibrio parahaemolyticus*	−	14	12	10
*Enterobacter cloacae*	−	15	12	8
*Escherichia coli*	−	15	10	6

Positive control: ciprofloxacin; negative control: discs without sample extracts.
